# Andrographolide suppresses cervical cancer progression by targeting angiogenesis and inducing apoptosis in a CAM-PDX model

**DOI:** 10.17305/bb.2025.11432

**Published:** 2025-02-11

**Authors:** Wanwan Zou, Jun Lou, Yun Yi, Yiming Cui, Xiaoyan Chu

**Affiliations:** 1 Department of Pathology, Jiangxi Provincial Cancer Hospital, Nanchang, China; 2 Department of Gynecological Oncology, Jiangxi Provincial Cancer Hospital, Nanchang, China; 3 Department of Mathematics, Applied Mathematics and Statistics, Case Western Reserve University, Cleveland, USA

**Keywords:** Cervical cancer, andrographolide, AND, angiogenesis inhibition, chorioallantoic membrane patient-derived xenograft, CAM-PDX

## Abstract

Cervical cancer poses significant clinical challenges, particularly in advanced stages. This study explores the therapeutic potential of andrographolide (AND), a bioactive compound derived from *Andrographis paniculata*, in mitigating cervical cancer progression using the chick embryo chorioallantoic membrane patient-derived xenograft (CAM-PDX) model. The model was validated through hematoxylin–eosin (H&E) staining and immunohistochemistry, which confirmed its ability to accurately replicate the histological and molecular characteristics of patient-derived xenografts (PDXs), establishing its reliability for therapeutic screening. A dose of 20 mg/kg AND was selected for further evaluation based on preliminary chorioallantoic membrane (CAM) assay findings. In the CAM-PDX model, AND significantly inhibited tumor growth, primarily by reducing angiogenesis and vessel density. Immunohistochemical analysis revealed that AND downregulated key proteins associated with cancer cell proliferation and survival, including Ki67, B-cell lymphoma 2 (BCL-2), and Erythroblast transformation-specific-related gene (ERG). These results indicate that AND not only disrupts tumor angiogenesis but also induces cell cycle arrest and promotes apoptosis in cervical cancer cells. In summary, this study successfully established a reproducible CAM-PDX model for drug evaluation and highlighted the potential of AND as a promising therapeutic candidate for cervical cancer, warranting further clinical investigation.

## Introduction

Cervical cancer, the second most common malignant tumor in women after breast cancer, ranks as the third leading cause of cancer-related deaths in women [1]. The prevalence of this life-threatening disease is gradually increasing worldwide, with approximately 18.1 million new cancer cases reported in 2018 [2]. While 60%–90% of patients diagnosed at an early stage of cervical cancer can be successfully treated, the survival rate for those with advanced or recurrent cervical cancer remains low, with a five-year survival rate of only 16.5% [3]. Several factors influence the development of cervical cancer, including exposure to human papillomavirus (HPV), deregulation of caspase enzymes, elevated expression of vascular endothelial growth factors (VEGF), overexpression of inhibitor apoptotic proteins (IAPs), and immune system failure [4]. Although treatments, such as chemoradiation and neoadjuvant therapy, are available, their effectiveness is often limited. This underscores the urgent need to explore more effective and innovative therapeutic agents. Andrographolide (AND), a diterpenoid lactone compound, has demonstrated a wide range of biological activities, including anti-tumor, immunomodulatory, anti-viral, anti-inflammatory, antibacterial, hepatoprotective, and cholagogic effects. In recent years, AND and its derivatives have garnered significant attention for their anti-tumor and anti-cancer properties. These compounds have shown promise in inhibiting the growth, proliferation, and migration of various cancer cell types [5]. However, the precise mechanisms by which AND exerts its effects on cervical cancer remain unclear. Considering the complexity of cervical cancer biology and the urgent need for effective therapeutic strategies, it is essential to use experimental models that closely replicate the disease’s characteristics. Cell-derived xenograft (CDX) and patient-derived xenograft (PDX) models are widely used in clinical and preclinical research; however, both have limitations in accurately predicting clinical outcomes [6]. These challenges have led to the development of more advanced and reliable cancer models. One such model, the chicken chorioallantoic membrane (CAM) model, has gained recognition in oncology research due to its cost effectiveness, rapid tumor formation, compatibility with diverse cell lines, and suitability for non-aseptic laboratory conditions [7]. CAM models have been successfully employed in research on various cancers, including ovarian, gastric, and prostate cancers, demonstrating their versatility and efficacy as experimental platforms [8, 9]. The highly vascularized CAM system effectively supports tumor xenograft development, enabling the study of angiogenesis-related mechanisms and the evaluation of anti-angiogenic drug efficacy. Chorioallantoic membrane patient-derived xenograft (CAM-PDX) models, which combine the strengths of PDX and CAM systems, offer reduced experimental costs alongside enhanced visualization and operability. Notably, these models retain the heterogeneity, pathophysiology, and key morphological and cytological features of the original tumors. Studies have reported an impressive success rate of 80%–100% in CAM-PDX models across various cancer types, including glioblastoma multiforme, sarcoma, and renal cell carcinoma [10, 11]. In this study, we utilized a chick embryo CAM xenograft model (CAM-PDX) to evaluate the therapeutic potential of AND in cervical cancer treatment. Our results demonstrate that AND effectively inhibits tumor growth and angiogenesis while promoting apoptosis in cervical cancer cells. This study highlights the potential of AND as a therapeutic agent and validates the CAM-PDX model as a reliable platform for further investigation into treatments for cervical cancer.

## Materials and methods

### Patient sample collection

This study received approval from the Ethics Committee of Jiangxi Cancer Hospital. A sample was collected from a 55-year-old female patient who reported postmenopausal irregular vaginal bleeding that began three months earlier. A cervical biopsy confirmed a diagnosis of squamous cell carcinoma (SCC), classified as stage IIA. Informed consent was obtained from the patient before sample collection. The tissue sample was promptly fixed in a 10% neutral formalin solution for subsequent staining.

### Nude mice and fertilized egg

Balb/c nude mice, aged 6–8 weeks, were obtained from GemPharmatech Co., Ltd. (Jiangsu, China) for the establishment and treatment of PDX models. The mice were maintained in a specific pathogen-free (SPF) environment. All animal studies were conducted with the approval of the Institutional Animal Care and Use Committee of Nanchang Royo Biotech Co., Ltd. (Approval No.: RYE2022081701). Fertilized eggs were obtained from Gallus domesticus (Ji’an, China), and AND was procured from Luye Biotechnology Company (Product No.: S24818-5g, China).

### Establishment of cervical cancer PDX models

Fresh SCC tissue from the patient was sectioned into small fragments measuring 2 mm × 2 mm × 2 mm and aseptically implanted subcutaneously into the scapulae of mice within 24 h. Once the tumor volume (P0) exceeded 1000 mm^3^, it was excised and re-implanted into other mice using the same method as the parental tumor. This process was repeated until the tumor was successfully propagated to the third generation (P3). This ensured that the tumoroid models retained the histopathological characteristics of the parental tumors while exhibiting transcriptional heterogeneity and diverse protein expression. Tumors from subsequent generations were then used for experiments. Tumor volumes were calculated using the formula: (length × width^2^)/2.

### Establishment of cervical cancer CAM-PDX models

**Figure 1.**
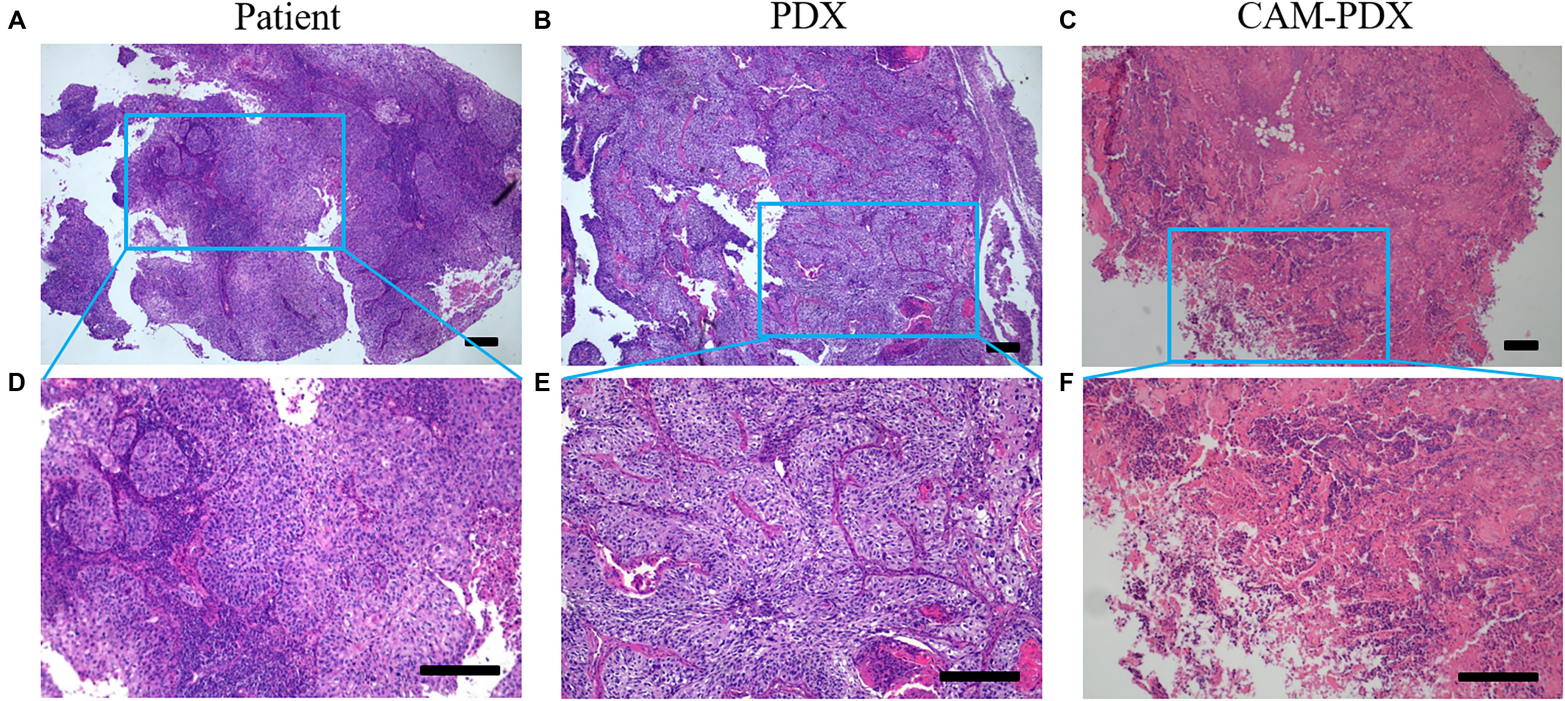
**Morphological comparison of patient-derived SCC, PDX, and CAM-PDX models using H&E staining.** (A–C) Low magnification images of the (A) patient tumor, (B) PDX model, and (C) CAM-PDX model; (D–F) High magnification images corresponding to the boxed regions in (A), (B), and (C), respectively. All models (PDX and CAM-PDX) replicate the pathological features of the patient’s tumor, including round cells, indistinct nucleoli, and scant cytoplasm. Scale bars, 100 µm. CAM-PDX: Chorioallantoic membrane patient-derived xenograft; PDX: Patient-derived xenograft; SCC: Squamous cell carcinoma; H&E: Hematoxylin and eosin.

Fertilized eggs were incubated until embryonic day 6 (ED6). On embryonic day 7 (ED7), small fragments of SCC tissue from the PDX model, measuring 3 mm × 3 mm × 3 mm, were implanted onto the CAM. The tumor fragments were positioned around the CAM’s blood vessels and secured in place using silicone rings. Drug treatments were administered within the silicone rings on embryonic days 10 and 13. On embryonic day 14, tumor tissues were harvested for drug efficacy evaluation (Figure 1). The study included four experimental groups: a nontreated control group (*n* ═ 6), a group treated with cisplatin (CDDP) at 2 mg/kg (*n* ═ 6), a group treated with AND at 20 mg/kg (*n* ═ 6), and a group treated with a combination of CDDP and AND (*n* ═ 6).

### Hematoxylin and eosin staining

Tumor tissues from the patient, PDX model, and CAM-PDX model were fixed in 10% neutral formalin for 24 h before being embedded in paraffin. The paraffin blocks were then sectioned into 4-µm thick slices using a microtome. These sections were mounted on glass slides and dried at 37 ^∘^C for at least 30 min. Subsequently, the slides were stained with hematoxylin and eosin (H&E) using an automatic slide stainer (DAKEWE Slide Stainer DP360 Series) following the manufacturer’s protocol. After staining, the slides were rinsed with distilled water, dehydrated through graded ethanol solutions, and cleared in xylene. Finally, the sections were coverslipped with mounting medium and prepared for microscopic examination.

### Immunohistochemistry

All specimens were sectioned from paraffin blocks and diagnosed using immunohistochemical staining one day after dewaxing. To block nonspecific binding, the sections were preincubated with 10% goat serum. Primary antibodies were then applied and incubated overnight at 4 ^∘^C: anti-Ki67 (Abcam, ab15580, 1:500 dilution, rabbit), anti-p53 (Abcam, ab26, 1:1000 dilution, mouse), and anti-erythroblast transformation-specific-related gene (ERG) (Abcam, ab92513, 1:5000 dilution, rabbit). Following primary antibody incubation, the tissue sections were treated with a biotinylated secondary antibody (Vector Labs) for 1 h at room temperature, then incubated with the avidin–biotin complex (ABC) reagent (Vector Labs) for 30 min. Peroxidase activity was visualized using a diaminobenzidine (DAB) kit (Vector Labs), resulting in a brown precipitate. The slides were counterstained with hematoxylin (Sigma) to visualize nuclei. Finally, the stained sections were examined and imaged using a Zeiss microscopic camera (Germany).

### CDDP and AND dose determination

First, 50 11-day-old non-transplanted chicken embryos were prepared and randomly divided into five groups, with ten embryos in each group. Each group received different doses of cisplatin, including a control group (normal saline) and doses of 2.0 mg/kg, 2.5 mg/kg, 3.0 mg/kg, and 3.5 mg/kg. The assigned dose of cisplatin was administered to the umbilical cord or choroid of each chicken embryo, with the time of administration recorded to ensure accurate dosing procedures. Following administration, the survival status of the embryos in each group was regularly monitored, and deaths were recorded. Embryos were considered alive if they showed normal activity within 48 h and were not otherwise deceased. Embryos were classified as dead if they exhibited no activity during observation at the time of opening and displayed turbid contents. The 50% lethal dose (LD50) of cisplatin was calculated using the modified Coriolis method through statistical analysis [12]. The dose of AND was selected based on standard medication guidelines and prior studies [13, 14].

Calculating LD50 (12):

**Figure 2.**
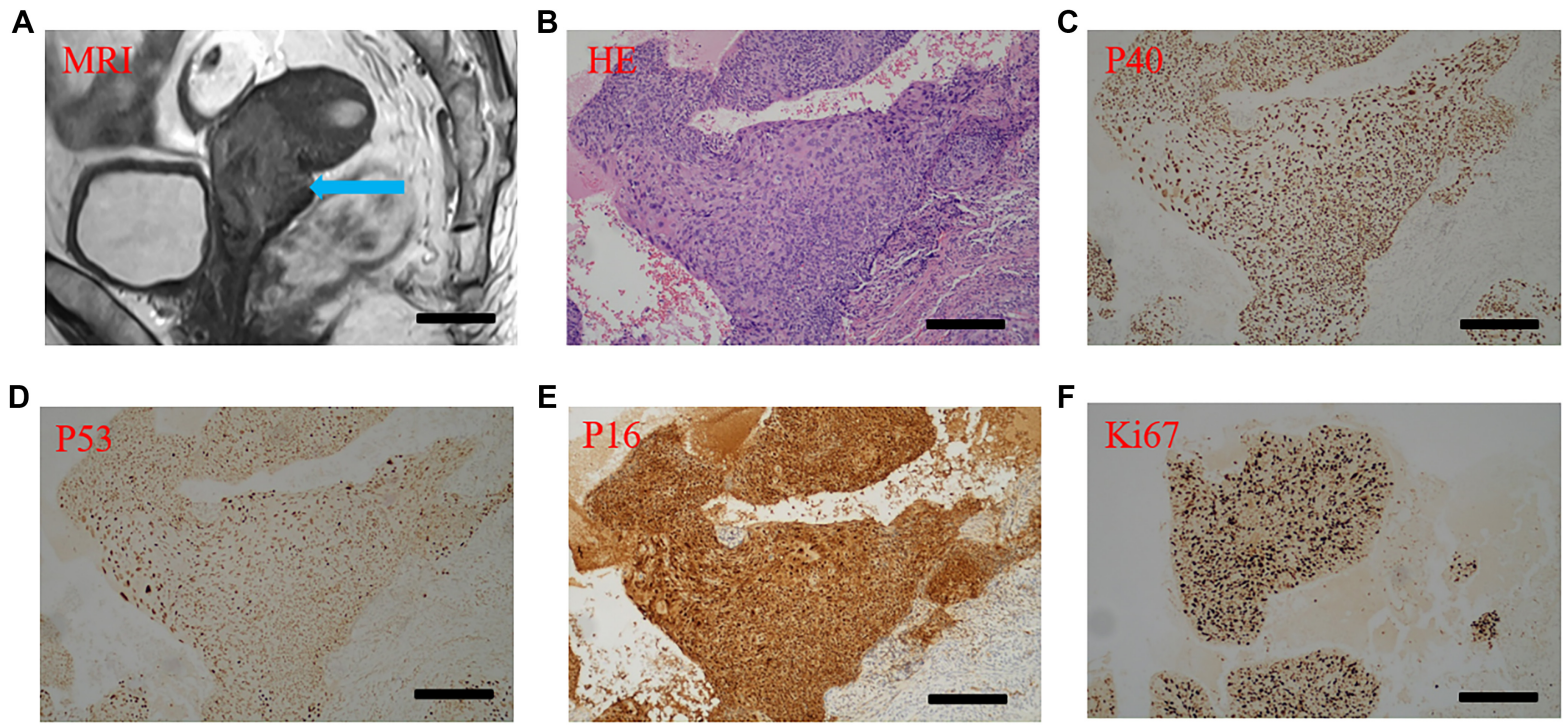
**Diagnostic imaging and histopathological analysis of the lesion.** (A) Sagittal T2-weighted MRI scan showing a well-defined mass (blue arrow); (B) H&E staining section displaying the histological architecture of the lesion; (C–F) Immunohistochemical staining of the lesion showing expression of various markers; (C) P40 staining; (D) P53 staining; (E) P16 staining; (F) Ki67 staining. Scale bars, 100 µm. H&E: Hematoxylin and eosin; MRI: Magnetic resonance imaging.

A ═ [ 50% − (% of mortality below 50%)] / [ (% of mortality above 50%) − (% of mortality below 50%)].

B ═ Log[(Dosage corresponding (to%) of mortality above 50%) / (Dosage corresponding (to%) of mortality below 50%)].

LD50 / embryo ═ Log−1 [Log (Dosage corresponding to% of mortality below 50%) + (A × B)].

### Ethical statement

The experimental protocols were approved by the Ethics Committee of the Jiangxi Provincial Cancer Hospital. Additionally, all animal experiments were reviewed and approved by the Institutional Animal Care and Use Committee of Nanchang Royo Biotech Co., Ltd. (Approval Number: RYE2022081701).

### Statistical analysis

Data were processed using SPSS 19.0 statistical software, and all results were analyzed with GraphPad Prism. Results are presented as means ± SEM. Comparisons among multiple groups were performed using two-way ANOVA, with a *P* value of < 0.05 considered statistically significant.

## Results

### The CAM-PDX model preserves the histopathological features of SCC

**Figure 3.**
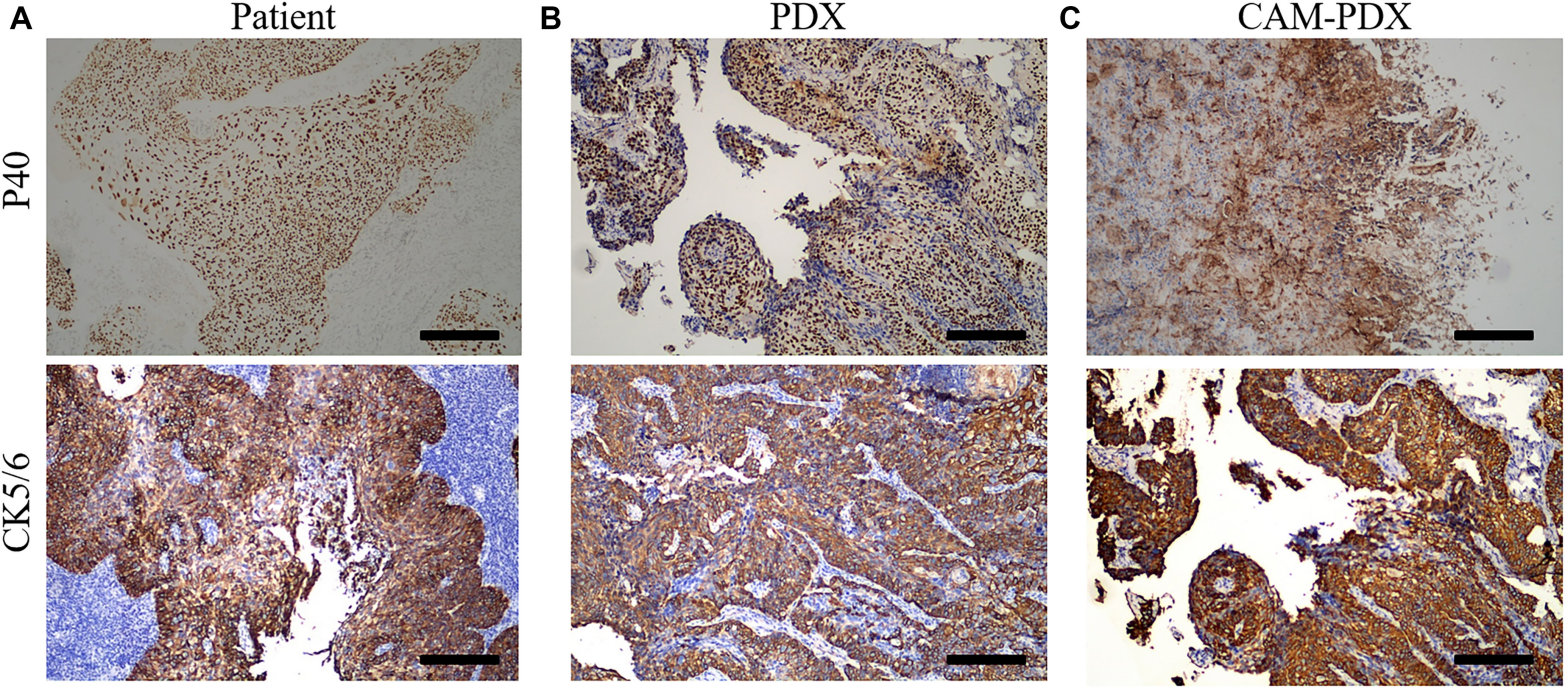
**Comparison of immunohistochemical P40 and CK5/6 in patient group, PDX group and CAM-PDX group.** (A) Patient tumor sample showing positive staining for both p40 and CK5/6, confirming the presence of these marker proteins; (B) PDX model sample demonstrating similar positive staining patterns for p40 and CK5/6, indicating retention of the patient’s protein expression profile; (C) CAM-PDX model sample also showing positive staining for p40 and CK5/6, further validating the similarity to the patient’s tumor. Scale bars, 100 µm. CAM-PDX: Chorioallantoic membrane patient-derived xenograft; PDX: Patient-derived xenograft.

Magnetic resonance imaging (MRI) of the patient revealed no evidence of distant metastases. Pathological analysis confirmed the presence of invasive keratinizing SCC, with tumor thrombi observed in the vessels. The depth of tumor invasion measured 1 cm, while the cervical thickness was approximately 1.6 cm. Notably, the left and right parametrium, vaginal resection margin, and pelvic lymph nodes were all negative for malignancy. Immunohistochemical analysis demonstrated positive expression of p40, p16, and p53 in the tumor tissues (Figure 2). The clinical diagnosis was consistent with SCC. To further investigate, we established both SCC PDX and CAM-PDX models, observing the morphological characteristics via H&E staining. The patient’s tumor exhibited a nest-like and flaky morphology, characterized by round cells, inconspicuous nucleoli, and sparse cytoplasm. Importantly, the PDX and CAM-PDX models displayed similar pathological morphologies to the patient’s tumor (Figure 1). Next, we evaluated the marker proteins p40 and CK5/6, which are associated with cervical SCC. p40, an isoform of p63, is typically highly expressed in squamous epithelial cells, while CK5/6, a member of the keratin family, is also prominently expressed in many squamous epithelial cells, particularly in SCC. The results showed that p40 and CK5/6 were consistently positive in the patient tumor, as well as in the PDX and CAM-PDX models. This finding reinforces the consistency of these models with clinical tumors and highlights their relevance in representing tumor phenotype, pathology, and potential treatment responses (Figure 3).

### Dose determination for CDDP and AND in CAM-PDX assay

**Figure 4.**
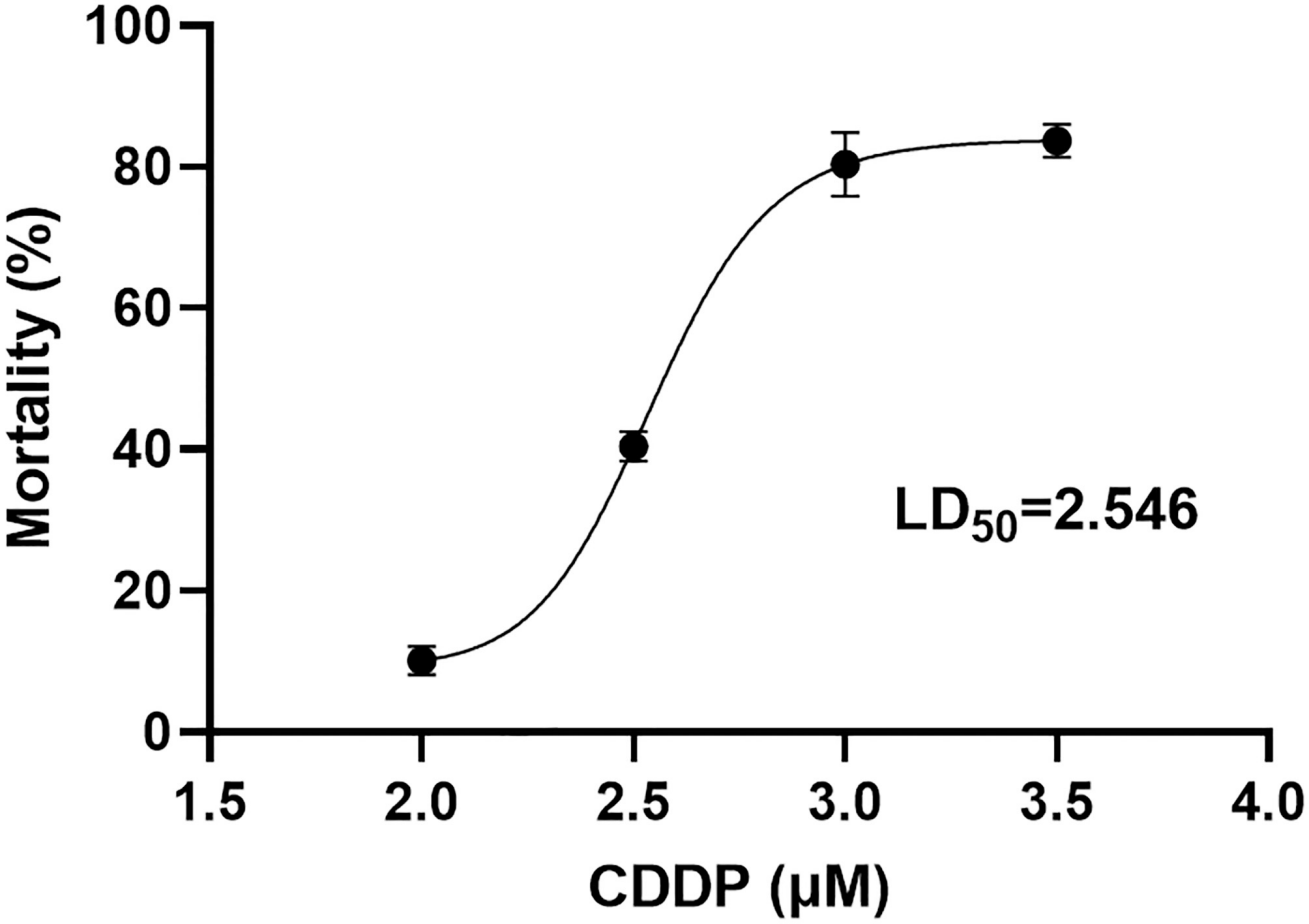
The LD50 values of 2.0 mg/kg, 2.5 mg/kg, 3.0 mg/kg, and 3.5 mg/kg CDDP in chicken embryos were calculated using the modified Coriolis method.

To determine the appropriate doses of CDDP and AND for the CAM-PDX assay, we treated 50 non-transplanted, 11-day-old chicken embryos, dividing them into five groups with varying doses of CDDP. Mortality rates were recorded, and the 50% LD50 was calculated using the modified Coriolis method [12]. The LD50 of CDDP was determined to be 2.546 mg/kg, with a 95% confidence interval of 2.542–3.036 mg/kg. Based on these findings, we selected a dose of approximately 2 mg/kg CDDP for subsequent experiments, adjusted according to the body weight of the chicken embryos (Figure 4). Additionally, a dose of 20 mg/kg AND was chosen based on standard medication guidelines [13]. These doses enable effective evaluation of the individual and combined effects of CDDP and AND in CAM-PDX models.

### AND inhibits angiogenesis in the CAM-PDX model of cervical cancer

**Figure 5.**
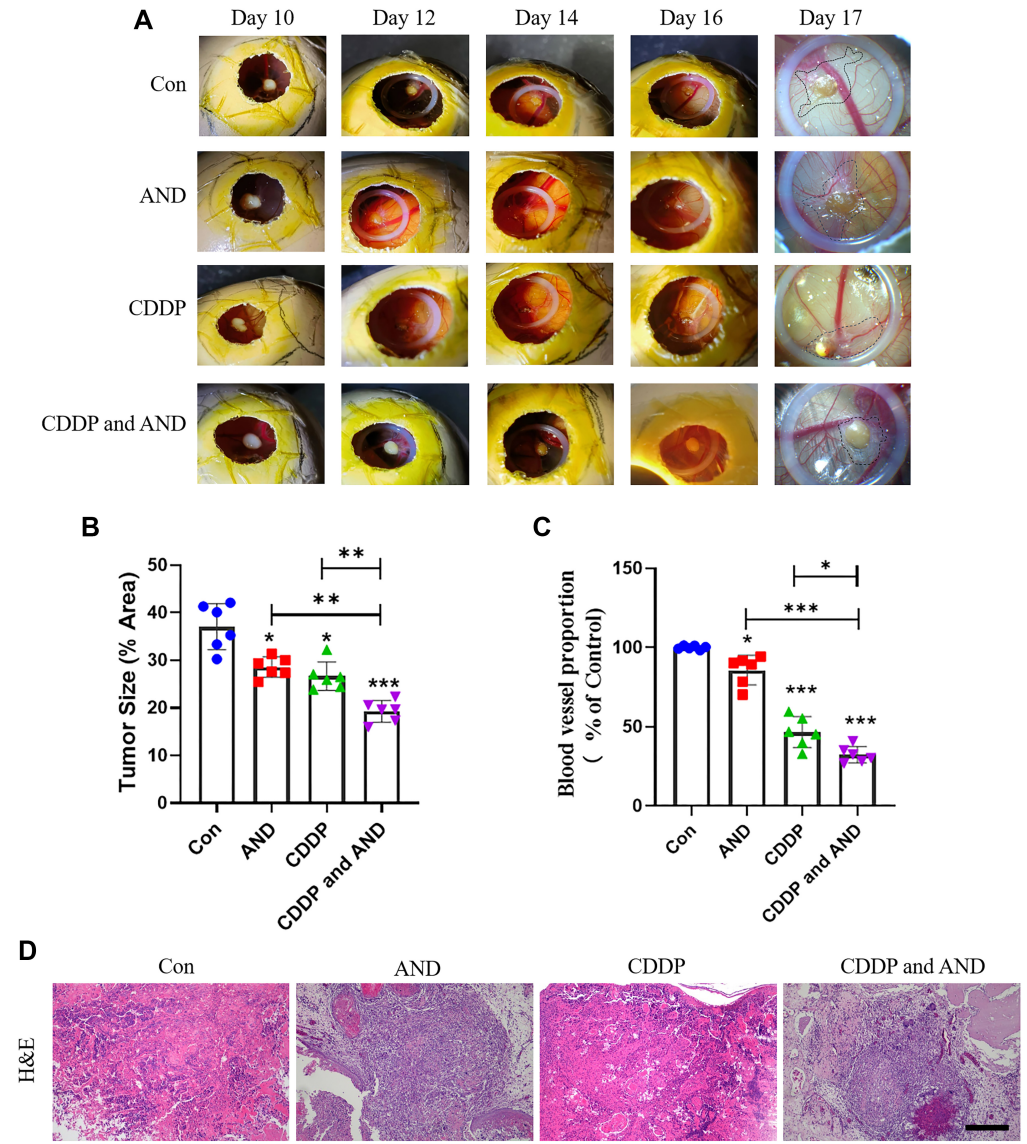
**Evaluation of tumor angiogenesis and morphology in CAM-PDX models.** (A) Angiogenesis assessment shows that untreated tumors exhibit extensive angiogenesis with large, converging blood vessels. AND treatment significantly inhibited vascularization and reduced small blood vessels. CDDP reduced tumor size and vessel density but had no additional dynamic changes with combined treatment with AND; Quantitative histograms show (B) tumor size and (C) angiogenesis rate in chick embryos treated with AND, CDDP, and AND+CDDP. Data represent mean ± SD of at least six independent experiments. *P < 0.05, **P < 0.01, ***P < 0.001. (D) H&E stain shows untreated tumor is poorly differentiated with dense nodules and fibrosis. AND has a better therapeutic effect, while CDDP treatment destroys the tumor structure, disperses tumor cells and causes tissue damage. Scale bars, 100 µm. AND: Andrographolide; CAM-PDX: Chorioallantoic membrane patient-derived xenograft; H&E: Hematoxylin and eosin.

To evaluate the therapeutic effects of AND, we assessed angiogenesis in the CAM-PDX model visually (Figure 5A). In the untreated control group, tumors exhibited irregular shapes and attracted numerous blood vessels, with new capillaries emerging perpendicularly from the tumor edge and converging at various angles. In contrast, tumors treated with 20 mg/kg AND showed a marked reduction in angiogenesis, characterized by the absence of large blood vessels and only a few small vessels extending into the tumor. Similarly, the CDDP-treated group demonstrated significant reductions in both tumor size and vascular density, indicating effective inhibition of tumor growth and angiogenesis. Notably, the combination of CDDP and AND yielded a more pronounced effect compared to monotherapy, with further reductions in tumor size and vascular density (Figure 5B and 5C). Histological examination of tumor biopsies is critical in clinical practice [15]. Therefore, we used H&E staining under light microscopy to evaluate tumor morphology in CAM-PDX models (Figure 5D). Tumors transplanted into the CAM-PDX model were poorly differentiated, with tumor cells displaying irregular, large, round, or oval shapes. Tumor nodules were surrounded by hyperplastic fibrosis and organized into nests or cords within stromal tissue. New blood vessels were observed around the tumors, with vessel density being higher at the infiltrated edges compared to the tumor core. Tumors treated with AND or CDDP exhibited significant disruption and disorganization of tumor architecture, leading to tumor cell dispersion and tissue damage. Importantly, tumors treated with the combination of CDDP and AND showed more extensive tissue disintegration, accompanied by greater reductions in vascularization and tumor growth. These results suggest that both CDDP and AND inhibit tumor cell proliferation and angiogenesis, with their combined use synergistically enhancing these effects.

### AND induces both cell proliferation and apoptosis in cervical carcinoma

**Figure 6.**
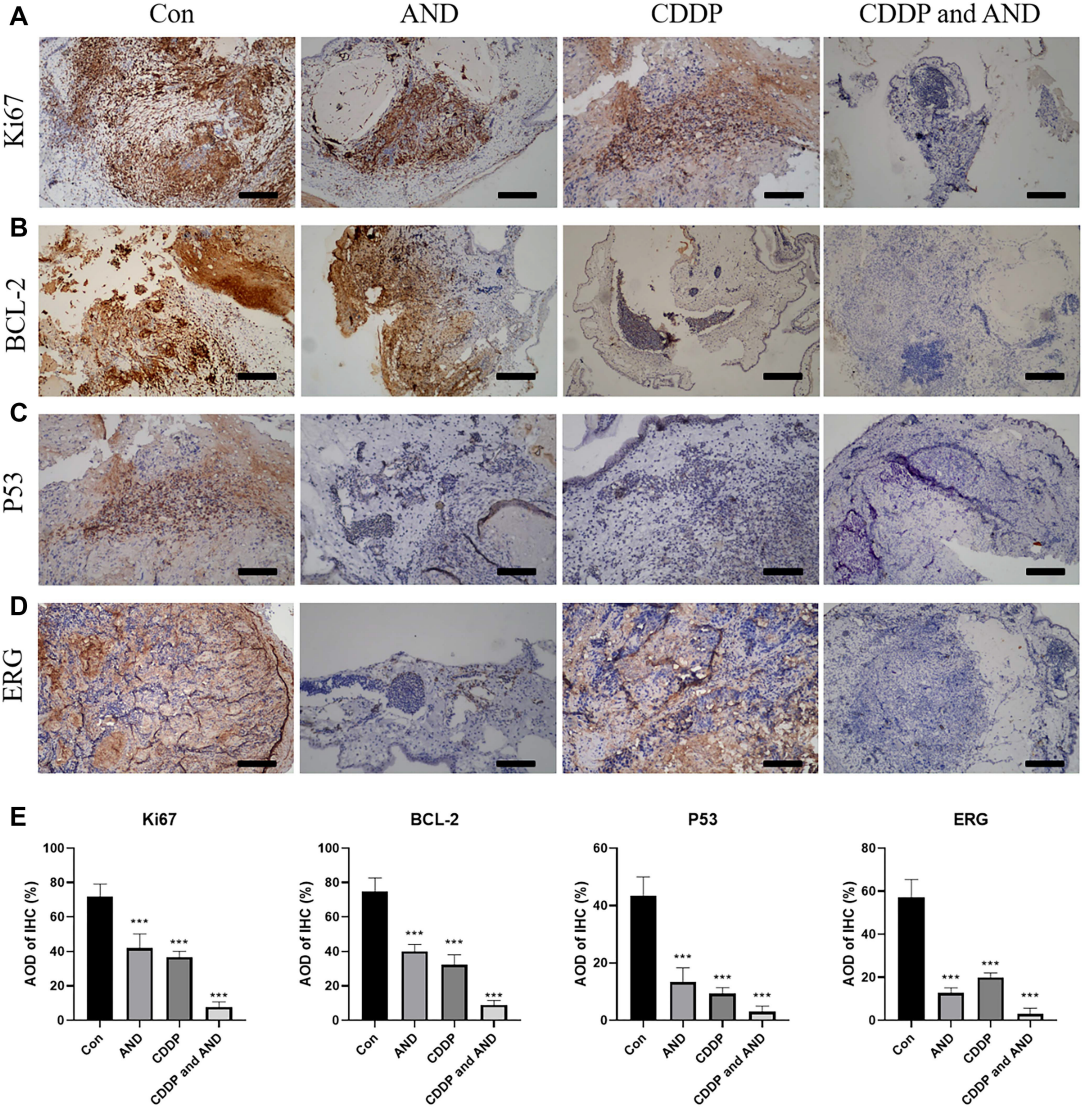
**Immunohistochemical analysis of tumor samples treated with different therapies.** IHC staining was performed to detect the expression of (A) Ki67, (B) BCL-2, (C) P53 and (D) ERG in xenografts after treatment in control, CDDP, AND and drug combination groups; (E) The average optical density (AOD) of Ki67, BCL-2, P53 and ERG was calculated by ImageJ. Untreated control tumors showed high levels of Ki67, p53, and ERG. While tumors treated with a combination of AND and CDDP showed significant reductions in Ki67, p53, and ERG accumulation. Scale bars, 100 µm. AND: Andrographolide; BCL-2: B-cell lymphoma 2; ERG: Erythroblast transformation-specific-related gene.

In our study, we evaluated the combined effects of AND and CDDP in xenograft models. The results demonstrated that the combination of AND and CDDP effectively inhibited tumor growth in mice, suggesting a potential synergistic effect in cancer treatment. To investigate the mechanism underlying this inhibitory effect, we performed an immunohistochemical analysis. Ki67, a marker of cell proliferation, is closely associated with cell division and proliferation activity [16]. We observed a significant reduction in Ki67 accumulation (*P* < 0.001) in the group treated with the combination of AND and CDDP, indicating that tumor cell proliferation was suppressed (Figure 6A and 6E). Additionally, we found that the combination treatment significantly reduced the expression levels of B-cell lymphoma 2 (BCL-2) and p53. These findings suggest that AND and CDDP may enhance therapeutic efficacy by promoting apoptosis and regulating tumor cell survival (Figure 6B, 6C, and 6E). ERG, a transcription factor implicated in tumor angiogenesis [17], was also significantly downregulated in the combination treatment group (Figure 6D and 6E). This result suggests that the combined treatment of AND and CDDP not only inhibits tumor growth but may also restrict the tumor’s nutrient and oxygen supply by suppressing angiogenesis, thereby further enhancing its therapeutic effect.

## Discussion

Cervical cancer is the most common gynecologic malignancy. In recent years, its incidence has shown a trend toward affecting younger individuals [18]. The disease has a high prevalence in developing countries and is primarily attributed to early sexual activity, infrequent condom use, and high-risk HPV infection. With the widespread implementation of cervical cytology screening, the early detection and treatment of cervical cancer and its precancerous lesions have significantly reduced both its incidence and mortality rates. Currently, the primary treatments for cervical cancer include surgery and radiotherapy, with chemotherapy serving as an adjunct in comprehensive treatment approaches [19, 20]. Most patients achieve a good prognosis, with a high 5-year survival rate. However, patients with lymph node metastases often face worse outcomes. Therefore, there is an urgent need to further investigate the pathogenesis of cervical cancer and develop novel therapeutic strategies. One promising advancement is the CAM-PDX model, which utilizes fertilized chicken eggs to implant patient-derived tumor specimens [21]. In our research, we optimized the growth conditions for cervical cancer tumors on the CAM in chicken embryos, which facilitated testing the effects of Chinese herbal compounds against cervical cancer. Moreover, the CAM-PDX models preserved the original tumor tissue structure, protein expression profile, cell proliferation characteristics, and apoptotic phenotype of the parental tumors. Notably, the feasibility of the CAM-PDX model as a testing platform for cervical cancer therapies was validated by demonstrating its concordance with clinical outcomes in patients receiving CDDP-based chemotherapy. This underscores the potential of CAM-PDX models in the development and validation of new treatment strategies for cervical cancer.

At the preclinical stage, developing low-cost, operational, and practical models can significantly accelerate the experimental process without compromising the accuracy or fidelity of disease models. The new animal model-building method described in our study offers a novel approach for quickly screening promising compounds for cervical tumors. Our results demonstrate that the CAM-PDX model provides a suitable tumor microenvironment that accurately simulates the growth characteristics of human tumors. The rapid construction of this model allows researchers to test tumor responses to chemotherapy and AND shortly after implantation. This streamlined preclinical study is far more efficient and cost-effective than the more complex and expensive mouse PDX studies. Consequently, our model offers an intriguing alternative for studying individual tumor biology and serves as an appropriate platform for testing various drugs in personalized oncology. Establishing a cervical cancer model using the CAM-PDX platform represents a highly valuable strategy, providing new opportunities for investigating mechanisms of drug action [22]. Recently, the antitumor effects of AND and its derivatives have drawn considerable attention. These compounds have been shown to inhibit the growth, proliferation, and migration of various cancer cells, including bladder cancer cells, prostate cancer cells, chronic myeloid leukemia cell lines, colon cancer cells, breast cancer cells, colorectal cancer cell lines, and non-small-cell lung cancer cells [23–25]. Many studies have highlighted AND’s role in inducing apoptosis and inhibiting growth [26]. Inhibiting tumor angiogenesis is an innovative strategy in cancer therapy. This study focused on the effect of AND on tumor angiogenesis, examining not only the dynamic changes in tumor angiogenesis using noninvasive microscopy but also cell proliferation, apoptosis, and angiogenesis using immunohistochemistry. Specifically, the expression of angiogenesis and cell proliferation biomarkers—Ki67, BCL-2, and ERG—was analyzed. It is well known that Ki67 is detectable during the G1, S, G2, and M phases of the cell cycle but is absent in the G0 phase, making it a reliable marker for proliferating cells [16]. After treatment with AND, Ki67 expression was reduced, suggesting that AND might exert its anticancer effects by inducing apoptosis. In addition, BCL-2, a key anti-apoptotic protein that regulates mitochondrial outer membrane permeabilization and cell survival [27], warrants attention. High BCL-2 expression is associated with resistance to apoptosis in various cancers, including cervical cancer. In this study, BCL-2 expression was high in the control group but decreased after drug treatment, particularly in the combination group. This finding suggests that AND may promote apoptosis by downregulating BCL-2 expression, thereby enhancing the efficacy of cancer cell apoptosis and offering a mechanistic explanation for its observed anticancer effects.

ERG is a member of the E-26 transformation-specific (ETS) transcription factor family. It serves as a marker of endothelial cell differentiation and plays a pivotal role in cell proliferation, differentiation, apoptosis, and angiogenesis. ERG demonstrates high sensitivity and specificity in endothelial vascular tumors, showing nuclear-positive expression, which makes it a reliable marker for diagnosing such tumors [17]. In this context, the down-regulation of ERG expression observed in cervical cancer tissues treated with AND suggests that AND inhibits cervical cancer progression by impairing blood vessel development and angiogenesis. Looking forward, further research is essential to investigate the broader therapeutic potential of AND in cervical cancer, particularly its influence on the tumor immune microenvironment. Exploring how AND modulates immune cell infiltration, regulates the expression of immune checkpoints, and activates immune responses within tumors could provide deeper insights into its antitumor mechanisms. These studies would help clarify whether AND can enhance antitumor immunity, positioning it as a candidate for combination therapies with immune checkpoint inhibitors or other immunotherapeutic strategies. Such research not only has the potential to expand our understanding of AND’s therapeutic benefits but also to support its clinical application in personalized cancer treatments.

## Conclusion

Overall, our results underscore the utility of the CAM-PDX models for evaluating therapeutic strategies and highlight the potential of AND in combination with CDDP for treating cervical SCC. Further research should focus on refining treatment regimens and investigating the mechanistic pathways driving the observed effects to further advance cervical cancer treatment.

## Data Availability

The datasets used and/or analyzed during the current study are available from the corresponding author on reasonable request
